# Compound sophora decoction alleviates ulcerative colitis by regulating macrophage polarization through cGAS inhibition: network pharmacology and experimental validation

**DOI:** 10.18632/aging.205734

**Published:** 2024-04-10

**Authors:** Fei Gao, Shuangjiao Deng, Yujin Liu, Pengcheng Wu, Lifen Huang, Feng Zhu, Chunzhu Wei, Yuyi Yuan, Yang Gui, Yushi Tian, Heng Fan, Hui Wu

**Affiliations:** 1 Department of Integrated Traditional Chinese and Western Medicine, Union Hospital, Tongji Medical College, Huazhong University of Science and Technology, Wuhan 430022, China; 2 Wuhan Central Hospital, Tongji Medical College, Huazhong University of Science and Technology, Wuhan 430022, China; 3 Department of Urology, Union Hospital, Tongji Medical College, Huazhong University of Science and Technology, Wuhan 430022, China

**Keywords:** compound sophora decoction, network pharmacology, ulcerative colitis, macrophage polarization

## Abstract

Introduction: Ulcerative colitis (UC) is a refractory disease with complex pathogenesis, and its pathogenesis is not clear. The present study aimed to investigate the potential target and related mechanism of Compound Sophora Decoction (CSD) in treating UC.

Methods: A network pharmacology approach predicted the components and targets of CSD to treat UC, and cell and animal experiments confirmed the findings of the approach and a new target for CSD treatment of UC.

Results: A total of 155 potential targets were identified for CSD treatment of UC, with some related to macrophage polarization, such as nitric oxide synthase (NOS2), also known as inducible nitric oxide synthase (iNOS). GO and KEGG enrichment analysis indicated that oxidative stress response and multiple inflammatory signaling pathways such as TNF-α may play a significant role. *In vitro* experiments revealed that Interferon-stimulated DNA (ISD) interference can cause polarization imbalances in Raw 264.7 and bone marrow-derived macrophages (BMDMs). Flow cytometry demonstrated that polarization of macrophages in the intestine, spleen, and lymph nodes *in vivo* was also unbalanced after dextran sulfate sodium (DSS) modeling with pathological intestinal injury. Both *in vitro* and *in vivo* studies indicated that after inducing inflammation, the levels of macrophage polarization-related markers (iNOS and Arg1) and inflammation-related factors (CCL17, IL10, TNF-α, and CXCL10) changed, accompanied by increased expression of cGAS. However, CSD treatment based on inflammation can inhibit the expression of cGAS protein and mRNA, lower the level of inflammatory factors, promote the expression of anti-inflammatory factors, and regulate macrophage polarization.

Conclusion: We concluded that CSD alleviated DSS-induced UC by inhibiting cGAS, thus regulating macrophage polarization.

## INTRODUCTION

Ulcerative colitis (UC), a subtype of inflammatory bowel disease (IBD), is a chronic inflammation of the colon mucosa characterized by lesions that extend from the rectum to a portion or even the entire colon. The incidence of UC is increasing yearly in newly industrialized countries. Continuous remission or relapse of UC seriously affects patients’ quality of life and places significant economic strain on national healthcare systems [1, 2]. The cumulative risk of colon cancer in UC patients increases as the disease progresses [3]. Current studies indicate diverse etiology of UC. The pathogenesis is complex and not fully understood, and commonly used pharmacologic agents (salicylates, immunosuppressants, and corticosteroids) primarily control symptoms but have little effect on preventing disease recurrence [4, 5].

Macrophages develop from monocytes in bone marrow, and when monocytes infiltrate tissues and organs, they fully differentiate into tissue-resident macrophages. Macrophages are associated with inflammatory diseases, and polarization imbalance is a significant cause of inflammation [6]. M1 and M2 macrophage phenotypes have pro-inflammatory and anti-inflammatory functions, respectively. The high expression of inducible nitric oxide synthase (iNOS) on the surface of M1 cells can produce inflammatory factors such as TNF-α and CXCL10, which can cause or aggravate intestinal inflammation. However, Arginase 1 (Arg1) is highly expressed on the surface of M2 cells, which can inhibit intestinal inflammation and repair intestinal damage, and produce anti-inflammatory factors such as IL-10 and CCL17 [7, 8]. In the intestinal tissues of UC patients and DSS-induced UC model mice, M1 cells are increased while M2 cells are decreased [9, 10]. Remodeling the balance of M1 and M2 cells can be used as one of the methods to reduce intestinal inflammation, while the method of restoring the balance and related mechanisms need to be investigated further.

After a pathogen enters the body, the pathogen’s DNA and the nuclear and mitochondrial DNA of the host cell damaged by the pathogen are released and accumulate in the cytoplasm of the host cell [11]. Cyclic guanosine monophosphate-adenosine monophosphate synthase (cGAS) is a cytoplasmic double-stranded DNA (dsDNA) sensor that effectively recognizes free cytoplasmic DNA, forms a complex to activate cGAS, and then produces the cyclic guanosine monophosphate-adenosine monophosphate (cGAMP) to trigger an innate immune response. Finally, the expression of type I interferon (IFN-β), TNF-α, and other inflammatory factors that can cause M1 cell polarization to improve the immune response [12, 13]. Interferon-stimulated DNA (ISD) transfection into cells can activate cGAS, inducing M1 polarization, whereas cGAS knockdown can promote microglial M2 polarization [14–16].

Traditional Chinese medicine (TCM) has irreplaceable advantages in the treatment of UC, which is primarily manifested in lower disease recurrence, fewer drug side-effects, and better repair of intestinal pathological damage than Western medicine [17–19]. UC belongs to these categories of TCM, blood stool, diarrhea, chronic dysentery, abdominal pain and intestinal abscess. TCM believes that the accumulation of dampness and heat in colon is the key pathogenesis of acute activity. CSD combines the traditional theory of TCM and clinical experience. The prescription composed of six Chinese herbs: Ku shen (*Sophora flavescens Aiton*), Di yu tan (*Sanguisorba officinalis* L.), Bai ji (*Bletilla striata* (Thunb.) Rchb.f.), Gan cao (*Glycyrrhiza glabra* L.), Qing dai (*Baphicacanthus cusia* (Nees) Bremek.), and San qi (*Panax notoginseng* (Burkill) F.H. Chen). The proportion is 15:15: 10:10: 3:3. Among them, Ku shen (*Sophora flavescens Aiton*) is the main medicine, first mentioned in *Shennong’s Herbal Classic*, clearing heat and drying dampness, being suitable for heat dysentery [20]. The efficacy and safety of Ku shen (*Sophora flavescens Aiton*) for ulcerative colitis have been confirmed in clinical studies [21]. According to *Pharmacopoeia of the People’s Republic of China* (*2020*), Di yu tan (*Sanguisorba officinalis* L.) and Qing dai (*Baphicacanthus cusia* (Nees) Bremek.) can clear heat and detoxify, cool blood to stanch bleeding for treating blood stool; The combination of Bai ji (*Bletilla striata* (Thunb.) Rchb.f.) and San qi (*Panax notoginseng* (Burkill) F.H. Chen) can dispel stasis to relieve swelling and pain, and astringe sores for treating abdominal pain; Gan cao (*Glycyrrhiza glabra* L.) can not only relieve abdominal pain but also reconcile drugs [20]. Therefore, the prescription has the power of clearing heat and drying dampness, detoxifying, astringing sores and generating muscle. Study has shown that CSD could treat UC [22]. In recent years, increasing evidence has been that TCM can regulate macrophage polarization to alleviate UC [23]. Therefore, the present study explored the effect of CSD on the polarization of UC macrophages and related targets using network pharmacology and experimental verification.

## MATERIALS AND METHODS

### Network pharmacological analysis

We used the same oral bioavailability (≥30%) and drug-likeness (≥0.18) thresholds that most researchers use to screen the active components of CSD in the Traditional Chinese Medicine System Pharmacology Database (TCMSP) [24]. The corresponding gene targets of these active components were then identified in TCMSP, and the UniProt knowledge database was used to transform these targets into *Homo sapiens* gene symbols. We sifted through UC-related disease genes from five databases, including GeneCards, DisGeNet, PharmGkb, Therapeutic Target Database (TTD), and DrugBank. The R software and scripts generated the Venn map of disease-drug-related genes. Based on this, Cytoscape software built the CSD regulatory network for UC treatment, and the STRING database constructed a protein-protein interaction (PPI) network with a high confidence score (≥0.9), manually integrating genes associated with macrophage polarization. The Gene Ontology (GO) and Kyoto Encyclopedia of Genes and Genomes (KEGG) pathway enrichment analyses were performed using R software to investigate the biological functions related to potential targets.

### Preparation of lyophilized powder and complete medium for CSD

The six herbs of CSD were obtained from Wuhan Union Hospital (Wuhan, China). All voucher specimens were deposited in the Laboratory of Integrated Tradition Chinese and Western Medicine, Union Hospital, Tongji Medical College, Huazhong University of Science and Technology (Wuhan, China) with numbers 2022011001, 2022011002, 2022011003, 2022011004, 2022011005 and 2022011006. We prepared CSD as before into a decoction [25]. The CSD decoction was stored at −80°C. The next day, we quickly placed it in a freeze-drying mechanism to produce lyophilized powder, which we sealed and stored at −20°C. One gram of lyophilized powder was dissolved into a 50 mL complete medium, stored at −80°C for a day, thawed, and centrifuged (3500 r, 10 min). The supernatant was then collected and filtered with a 0.22 μm filter to obtain a drug-containing medium with a concentration of 20 mg/mL [26].

### Cell

Mouse tibiofibula were rinsed to prepare bone marrow mesenchymal cell suspension, and cells were stimulated with M-CSF (30 ng/mL) for seven days to induce their differentiation into mouse BMDMs. Cell morphology was observed seven days later, and BMDMs were identified using flow cytometry. The murine RAW264.7 macrophage cell line was purchased from WARNER and incubated at 37°C in a 5% CO_2_ incubator. Transfection of ISD (2 μg/mL) with Lip3000 was used to establish an inflammation model of BMDMs. Xiang Gui et al. [15] described the preparation method of ISD. Cell viability is measured using Cell Count Kit-8 (CCK-8, Biosharp, Hefei, China).

### Animals and experimental protocols

Male C57BL/6J mice (weighing 20–22 g and aged 6–8 weeks) were purchased from SPF Biotechnology Co., Ltd. (Beijing, China); (Quality certification: SCXK (jing) 2019-0010). Mice were raised at the Experimental Animal Center of Huazhong University of Science and Technology (HUST, Wuhan, China). The mice had free access to feed and water under the specific pathogen-free (SPF) condition and were fed adaptively for five days. The mice were divided into three groups (*n* = 8), normal control group (Control), model group (DSS), and CSD group (14.56 g/kg). Acute colitis was induced by 3% DSS. CSD was gavaged for seven days from the start of the modeling, and the model group with the same amount of drinking water. Mice were sacrificed on the eighth day, and spleen, mesenteric lymph nodes, and intestinal tissues were collected.

### Evaluation of animal inflammation

Starting from the day before modeling, we regularly monitored the weight loss, fecal morphology, blood status in stool, and activity of mice every day until the end of the experiment. The disease activity index (DAI) is calculated based on the severity of symptoms [27]. The intestinal tract of mice was measured after sacrifice, and the intestinal tissue near the anus was transected for Hematoxylin and Eosin (H&E) and Alcian Blue/Phosphoric Acid Schiff (AB/PAS) staining, which were used to assess the degree of pathological damage of intestinal tissue and goblet cell destruction, respectively.

### Immunofluorescent (IF)

RAW264.7 cells were fixed with 4% paraformaldehyde after treatment and incubated overnight (4°C) with anti-iNOS (1:200) and anti-Arg1 (1:500). The next day, anti-iNOS was incubated with fluorescein isothiocyanate (FITC) fluorescence secondary antibody, while anti-Arg1 was with Cy3. After dewaxing and dehydrating the paraffin sections of intestinal tissue, the antigen was repaired with citrate buffer and sealed with goat serum. The sections were incubated overnight with anti-dsDNA-antibody (1:200). Before adding 4′,6-diamidino-2-phenylindole (DAPI), the secondary antibody was incubated for 1 h. Finally, an anti-fluorescence quencher was added, and we could observe the fluorescent expression of cells using a fluorescence microscope (Olympus, Tokyo, Japan).

### Western blotting (WB) and enzyme-linked immunosorbent assay (ELISA)

The protein concentration was determined by Bicinchoninic Acid (BCA) assay after protein extraction from mouse intestinal tissue and RAW264.7, and the dosage was determined based on the concentration. Primary antibodies include anti-cGAS (1:2000), anti-β-actin (1:1000), anti-GAPDH (1:2000), anti-α-Tubulin (1:2000,), anti-iNOS (1:1000), and anti-Arg1 (1:5000). ImageJ Software was used to quantify WB. The expression levels of IL10, CCL17, CXCL10, and TNF-α in intestinal tissues were quantified by ELISA kits in accordance with the manufacturer’s instructions.

### Real-time polymerase chain reaction (PCR)

Total RNA was extracted from cells and tissues following the manufacturer’s instruction. It was then reversely transcribed into cDNA with reverse transcription reagent, and cDNA was amplified using an amplification kit. Supplementary Table 1 lists all mRNA primers.

### Flow cytometry

BMDMs were stained, and mononuclear cells were isolated from the intestinal tract, spleen, and mesenteric lymph nodes. The spleen and mesenteric lymph nodes of mice were thoroughly cut up in phosphate buffer saline (PBS) solution containing 2% Bovine Serum Albumin (BSA), strained through a 70 μm strainer, and suspended after centrifugation. A digestive fluid containing Collagenase IV (2 mg/mL) and DNase-I (0.25 mg/mL) was used to digest the intestinal tissue. The digestive fluid was placed in a 38°C water bath for 40 min. Every 5 min, the lid was opened and shaken to aid digestion. Following digestion in a 1640 base medium, an intestinal lamina propria cell suspension was prepared using a 70 μm strainer.

Flow cytometry antibodies, including BV510-conjugated anti-FVD antibody, APC-Cy7-conjugated anti-CD45 antibody, PE-conjugated F4/80 antibody, PC-CY5.5-conjugated CD11b antibody, BV421-conjugated CD11C antibody, and APC-conjugated CD206 antibody were incubated at 4°C for 20 min against light. The stained cells were analyzed by flow cytometry after washing.

### Intestinal barrier function assays

The abdominal hair of mice was removed after 1% pentobarbital anesthesia. After fasting for 24 h and keeping mice away from light, FITC-Dex (average molecular weight: 4000, 0.6 mg/g) was administered intragastrically. Fluorescence intensity (excitation 490 nm/emission 535 nm) was measured in 4 h. The 80 μL of serum was taken and then diluted in a ratio of 1:1 with PBS solution. The FITC-Dex content in serum was measured using a fluorescent enzyme label according to the manufacturer’s instructions.

### Statistical analysis

The data were presented as mean ± standard deviation (Mean ± SD), with n representing the number of independent experiments. The unpaired two-tailed Student *t*-test was used to analyze data from two groups. Datasets including data from more than two groups were analyzed with One-way ANOVA with Bonferroni post hoc test, and only results with *p* < 0.05 were considered statistically significant. GraphPad Prism 8 software (version 8) was used for statistical analysis.

### Data availability statement

Data will be made available on request.

## RESULTS

### Screening the components of CSD in the treatment of UC and macrophage polarization-related targets and signaling pathways

**Figure 1:**
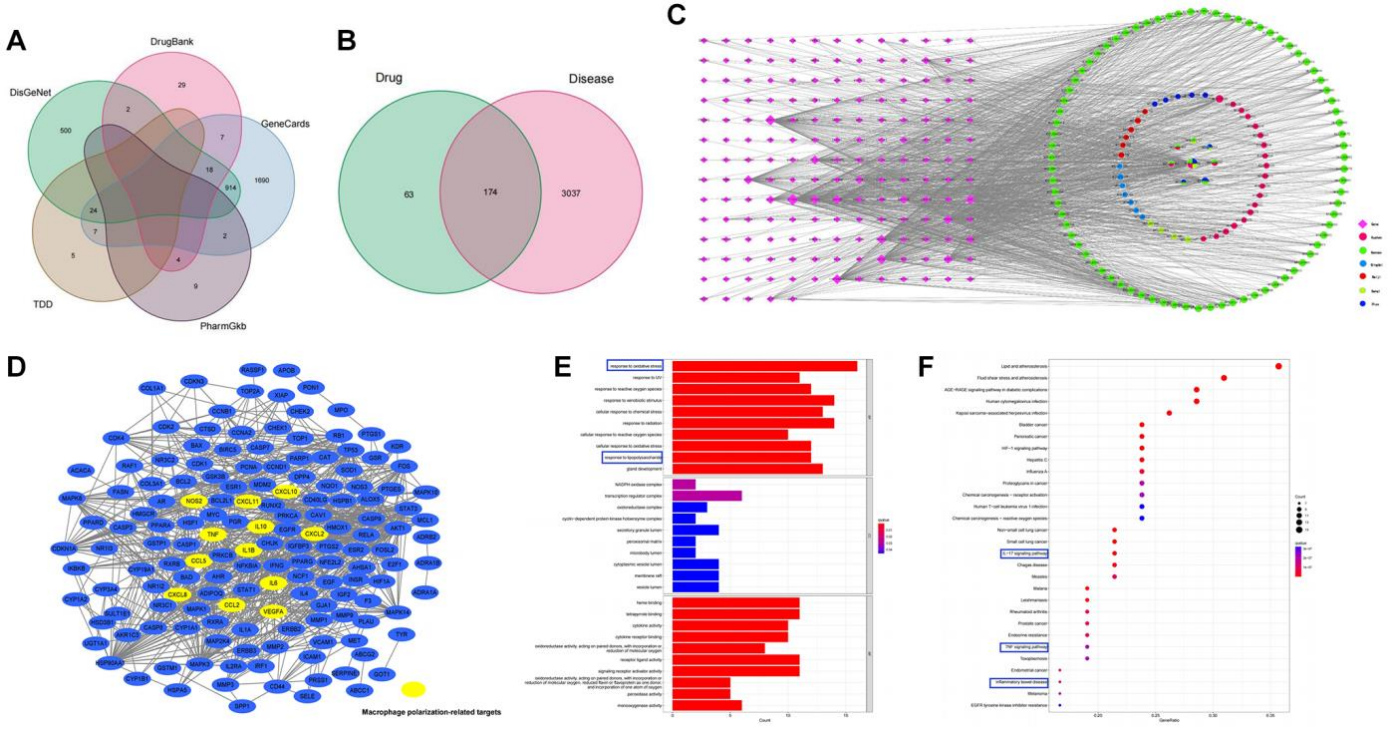
**Pharmacology analysis of CSD.** (**A**) Venn diagram of UC-associated genes in five databases; (**B**) Venn diagram of CSD target genes and differentially expressed genes in UC; (**C**) The CSD–active compound–target network; (**D**) Genes associated with macrophage polarization in PPI network; (**E**) GO analysis of CSD-targeted genes (*p* < 0.05); (**F**) KEGG analysis of CSD-targeted genes.

A total of 165 active components were screened from six TCMs of CSD in TCMSP, and eight of them were present in multiple medicines, with quercetin being present in four TCMs (Ku shen, Di yu tan, San qi, and Gan cao), indicating that quercetin was one of the main active components of CSD (Supplementary Table 2). Potential targets corresponding to these active components in TCMSP, in combination with 3211 UC-associated targets in five disease gene databases (Figure 1A), resulted in the identification of 174 UC-CSD-associated targets (Figure 1B). The Cytoscape software was used to establish the compound-target relationship network (Figure 1C).

A PPI network was obtained after importing 174 genes into STRING (Figure 1D), and the targets associated with macrophage polarization were manually labeled. The genes associated with M1 macrophage polarization included NOS2 (iNOS), CXCL10, TNF-α, IL-6, CCL5, CXCL8, CXCL2, and CXCL11. Whereas IL10, VEGF-α, IL-1β, and CCL2 were the genes associated with M2 macrophage polarization (Figure 1D). We performed GO and KEGG analysis on the target genes to further investigate the mechanism of CSD in treating UC. The results revealed that these genes were enriched in the following aspects: oxidative stress response, lipopolysaccharide response, IL-17 signaling pathway, TNF-α signaling pathway, and IBD. The first ten items of biological processes (BP), cell components (CC), and molecular functions (MF) that were significantly enriched in GO analysis were displayed (Figure 1E), as were the first 30 items of KEGG analysis (Figure 1F). The therapeutic effect of CSD on UC was reflected in the regulation of inflammation and oxidative stress.

### Inducing BMDMs successfully and determining the intervention concentration of CSD *in vitro*

**Figure 2:**
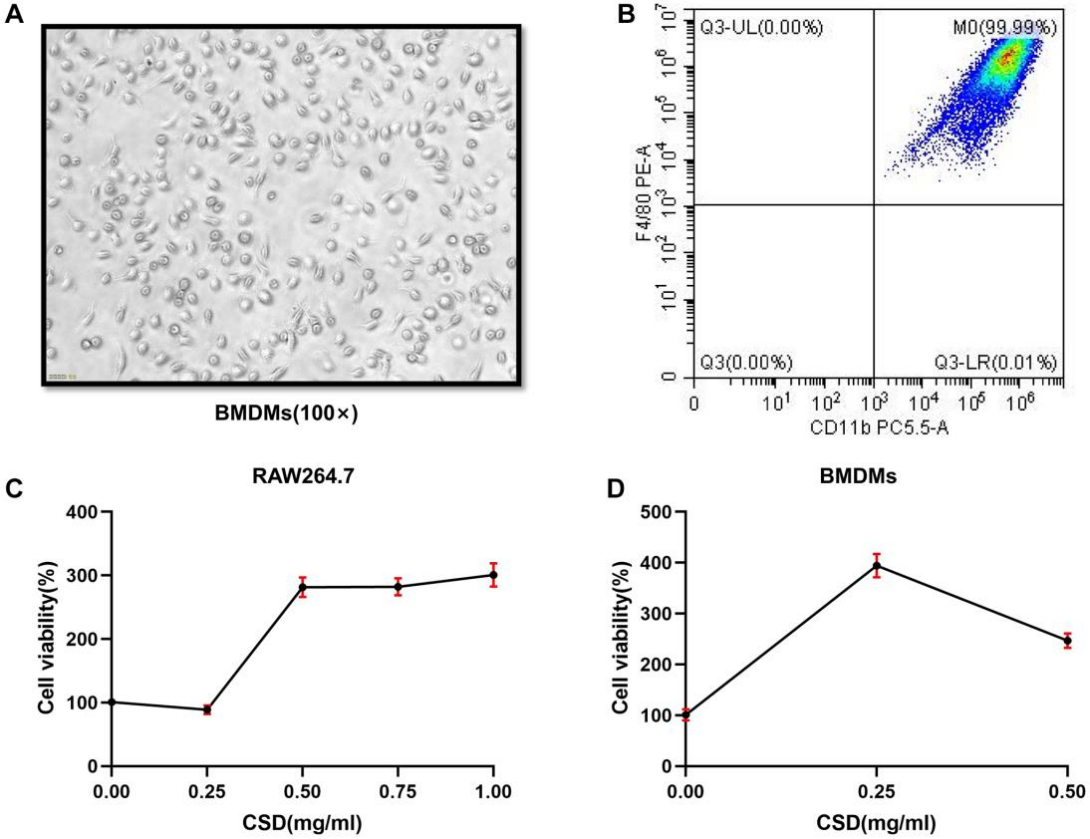
**Inducing BMDMs successfully and determining the intervention concentration of CSD *in vitro*.** (**A**) Morphology of BMDMs at the seventh day of induction, 100X magnification; (**B**) Flow cytometry figure of BMDMs identification; (**C**) CCK8 assay of RAW264.7 cells interfered by CSD for 6 h; (**D**) CCK8 determination of CSD intervention in BMDMs for 6 h. The data represent the mean ± SD of three independent experiments.

BMDMs and RAW264.7 cells were used for experiments to study *in vitro* macrophage polarization. First, identifying the induced BMDMs using the microscope on the seventh day of induction, the cell morphology was fusiform with two open ends (Figure 2A). Flow cytometry revealed that the proportion of CD11b+F4/80+ cells reached 99.99% (Figure 2B), indicating successful induction of BMDMs [28]. Count Kit 8 (CCK8) test was then performed on RAW264.7 cells to test cell viability. The findings revealed that CSD did not significantly reduce the cell viability at 0.25 mg/mL and 0.5 mg/mL for 6 h after intervention (Figure 2C), nor did it affect the cell viability in BMDMs (Figure 2D). Therefore, 0.25 mg/mL was selected as the low-dose CSD (CSDL) group and 0.5 mg/mL as the high-dose (CSDH) group.

### CSD restoring macrophage balance *in vitro*

**Figure 3:**
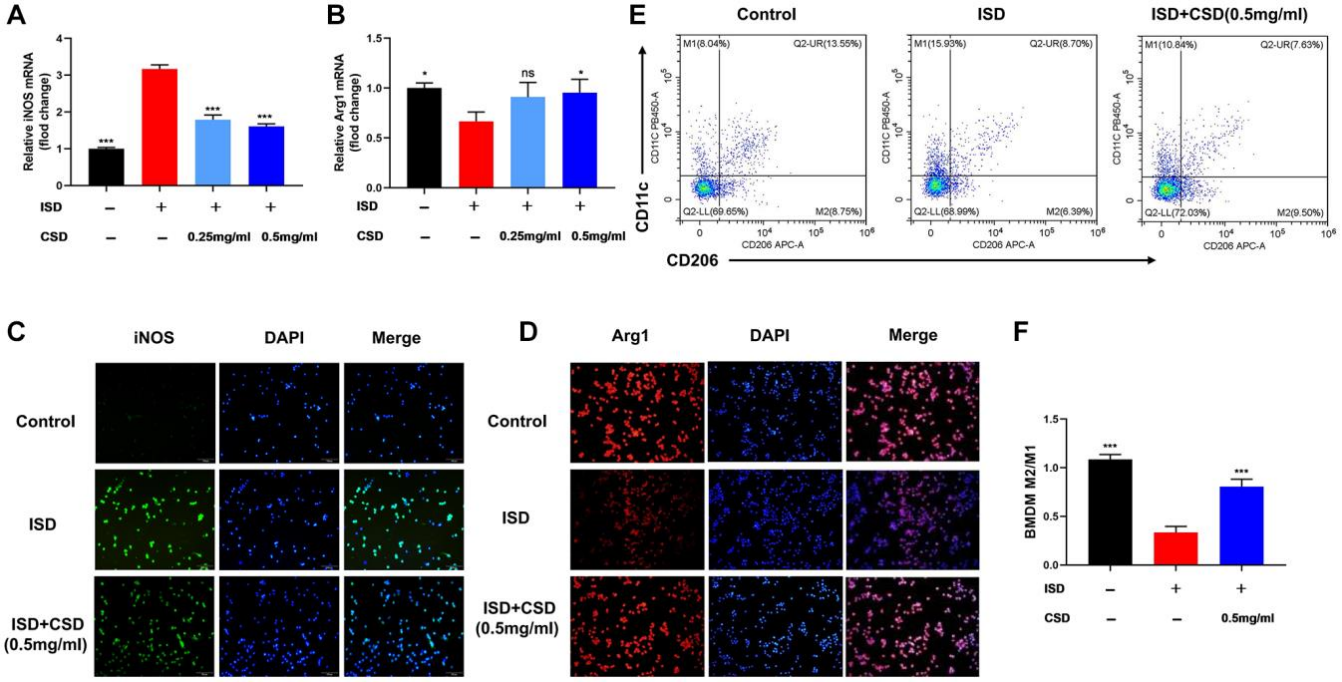
**CSD restores macrophage balance *in vitro*.** (**A**) iNOS mRNA levels in RAW264.7 cells; (**B**) Arg1 mRNA levels in RAW264.7 cells; (**C**) Fluorescence expression of iNOS in RAW264.7 cells, 100X magnification; (**D**) Fluorescence expression of Arg1 in RAW264.7 cells, 100X magnification; (**E**) Flow cytometry diagram of M1 and M2 in BMDMs; (**F**) M2/M1 cell ratio in BMDMs. The data represented as mean ± SD of three independent experiments. Abbreviation: ns: no significance. ^*^p < 0.05, ^***^p < 0.001 vs. ISD group.

*In vitro* experiments were performed first to confirm the effect of CSD on macrophage polarization. iNOS is an M1 macrophage marker, while Arg1 is an M2 macrophage marker [29]. After ISD stimulation, the level of iNOS mRNA increased while Arg1 mRNA decreased in RAW264.7 cells. However, the addition of CSD intervention could reverse this effect, particularly at a concentration of 0.5 mg/mL (Figure 3A, 3B). The expression of iNOS and Arg1 in cell fluorescence assay was identical (Figure 3C, 3D). Flow cytometry also revealed that the M2/M1 ratio of BMDMs decreased after ISD induction but increased after CSD addition (Figure 3E, 3F). These findings suggested that CSD could regulate ISD-induced macrophage polarization *in vitro*.

### CSD inhibits cGAS in macrophages and regulates cellular inflammation *in vitro*

**Figure 4:**
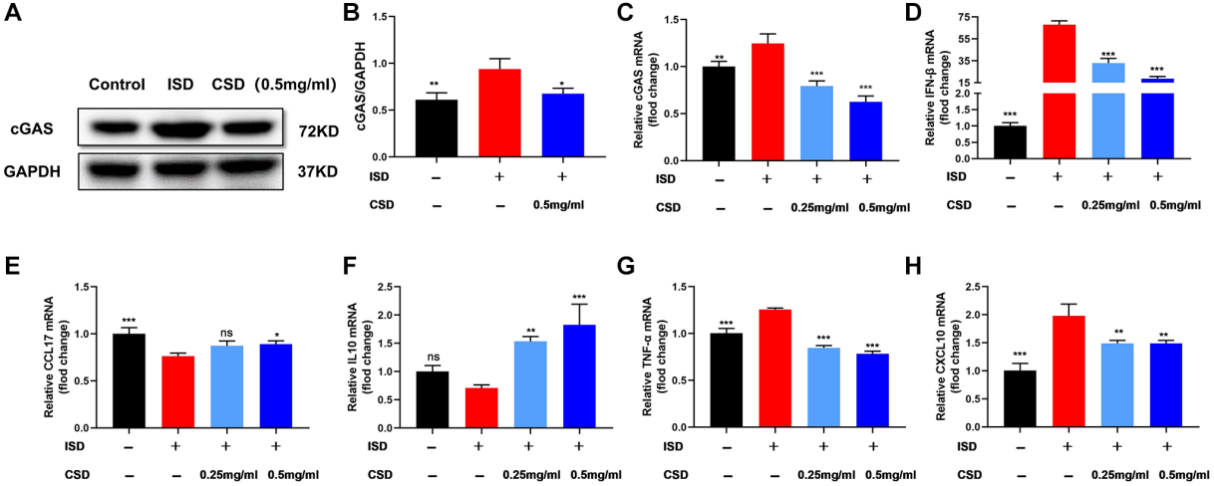
**CSD inhibits cGAS in macrophages (RAW264.7) and regulates cellular inflammation *in vitro*.** (**A**) Representative western blot of cGAS; (**B**) Statistical map of cGAS protein content; (**C**) cGAS mRNA levels; (**D**) IFN-β mRNA levels; (**E**) CCL17 mRNA levels; (**F**) IL10 mRNA levels; (**G**) TNF-α mRNA levels; (**H**) CXCL10 mRNA levels. The data present the mean ± SD of three independent experiments. Abbreviation: ns: no significance. ^*^p < 0.05, ^**^p < 0.01, ^***^p < 0.001 vs. ISD group.

We detected the expression of cGAS, a protein associated with macrophage polarization, to further explore the mechanism by which CSD regulates macrophages. Findings of WB and PCR revealed that ISD induced the expression of cGAS protein and mRNA in RAW264.7 macrophages, and the expression decreased after CSD intervention (Figure 4A–4C). IFN-β is an inflammatory factor in the complex downstream pathway of cGAS [30], and its mRNA level is correlated with cGAS expression (Figure 4D). The mRNA levels of CCL17, IL10, TNF-α, and CXCL10 were used to assess ISD-induced macrophage inflammation. The results revealed that ISD decreased the mRNA levels of anti-inflammatory factors CCL17 and IL10 while increasing the inflammatory factors TNF-α and CXCL10, and CSD changed the outcome (Figure 4E–4H). These findings imply that CSD may regulate macrophage polarization and inhibit inflammation by inhibiting cGAS.

### CSD alleviated symptoms and intestinal damage in UC mice

**Figure 5:**
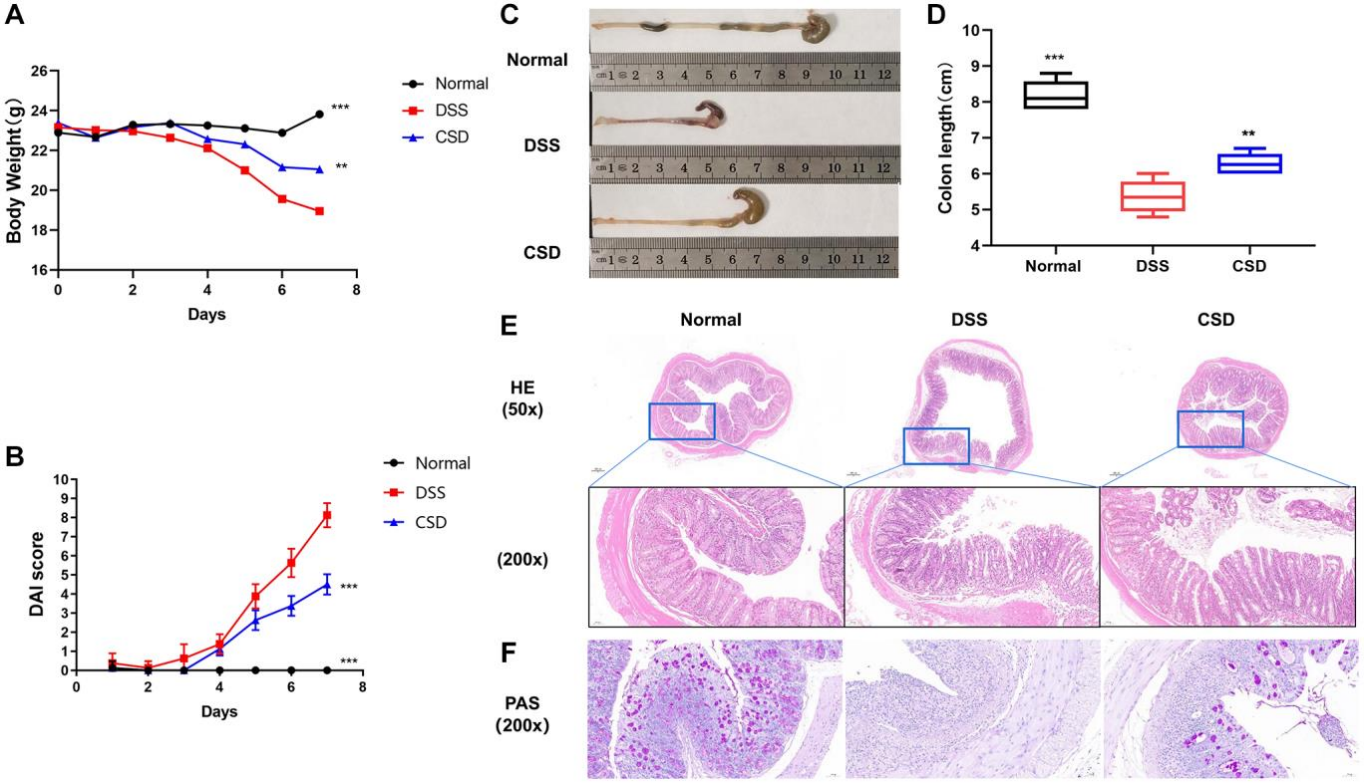
**CSD alleviated symptoms and intestinal damage in UC mice.** (**A**) Changes in body weight of mice; (**B**) Images of the colons of mice; (**C**) Statistical map of colon length in mice; (**D**) DAI score of mice; and (**E**) Mouse intestinal HE staining. (**F**) Mouse intestinal PAS staining. The results are mean ± SD, *n* = 6–8, ^**^p < 0.01, ^***^p < 0.001 vs. DSS group.

A DSS-induced colitis model was established to study the effects of CSD on the symptoms and pathological changes of UC mice, and CSD was given by gavage on this basis. CSD treatment of DSS-induced UC in mice reduced intestinal bleeding, stabilized stool traits, and increased body weight (Figure 5A). It decreased cumulative (DAI) score (Figure 5B). Inflammation can shorten the colon. The colon length increased significantly after CSD treatment compared to the DSS group (Figure 5C, 5D). In DSS-induced mice, HE staining revealed intestinal pathological injury, intestinal structure destruction, and increased inflammatory infiltration (Figure 5E). The observation of changes in goblet cell number was an important morphological basis for intestinal structural changes [31]. AB/PAS staining demonstrated a depletion in the number of intestinal goblet cells in the DSS group compared to the control group. However, this depletion was reduced after CSD intervention (Figure 5F). These findings imply that CSD could alleviate the progression of DSS-induced UC.

### CSD decreased the inflammatory phenotype and increased the anti-inflammatory phenotype of macrophages in UC mice

**Figure 6:**
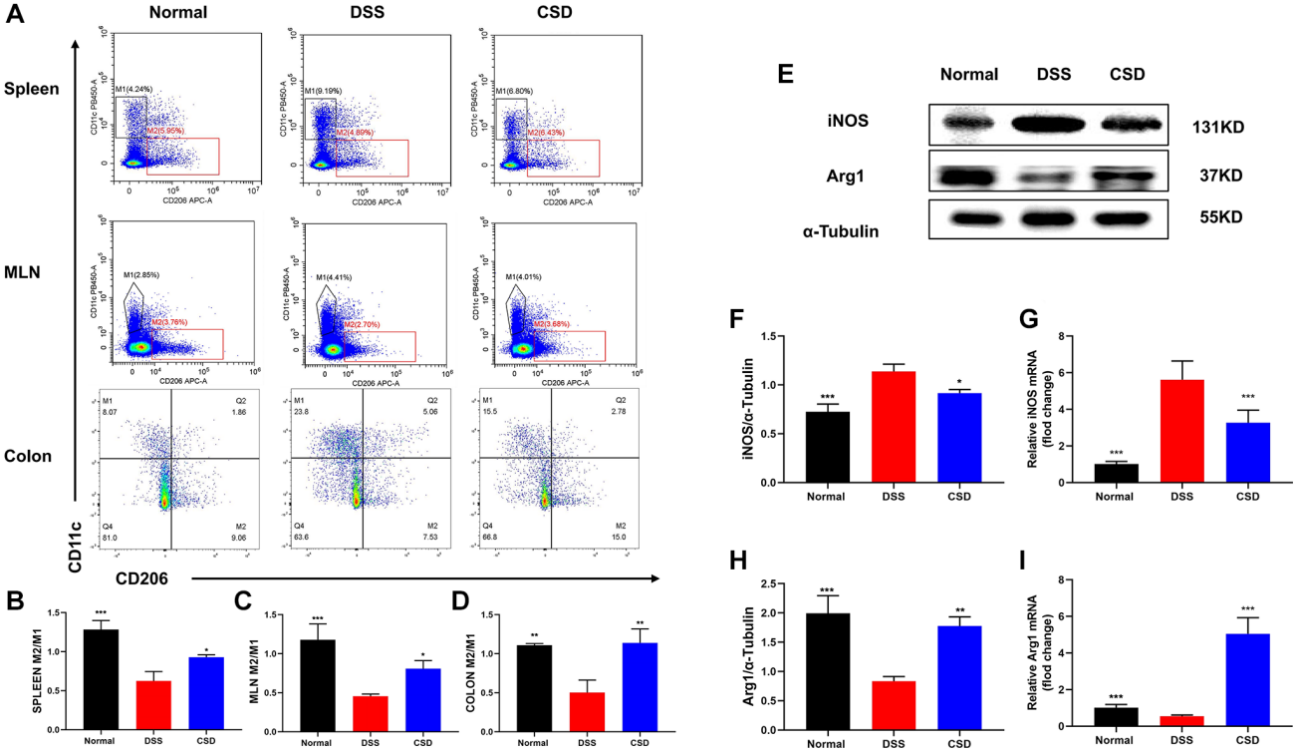
**CSD-regulated macrophage polarization *in vivo*.** (**A**) Flow cytometry diagram of M1 and M2 in the spleen, mesenteric lymph nodes, and intestine of mice; (**B**) M2/M1 ratio of spleen in mice; (**C**) M2/M1 ratio of mesenteric lymph nodes in mice; (**D**) M2/M1 ratio of intestine in mice; (**E**) Representative western blot of iNOS and Arg1 in intestine; (**F**) Statistical map of iNOS protein content in intestine; (**G**) iNOS mRNA levels in intestine; (**H**) Statistical map of Arg1 protein content in the intestine; (**I**) Arg1 mRNA levels in intestine. The results are mean ± SD, *n* = 3–6, ^*^p < 0.05, ^**^p < 0.01, ^***^p < 0.001 vs. DSS group.

The spleen, mesenteric lymph nodes, and intestine are all important immune system components, and the UC lesion site is in the intestine. Therefore, we focused on detecting macrophage polarization in these sites to investigate the effect of CSD on UC mice. Compared to the control group, DSS could induce macrophages to differentiate into inflammatory phenotypes (M1), and M2/M1 decreased, whereas CSD significantly increased this ratio (Figure 6A–6D). We measured the protein and mRNA levels of iNOS and Arg1 in intestinal tissues, indicating that CSD could lower the content of iNOS while increasing Arg1 in intestinal tissues after DSS-induced UC (Figure 6E–6I). These findings suggest that CSD regulates DSS-induced macrophage polarization in UC mice.

### CSD inhibited the expression of cGAS and the content of inflammatory factors in the intestinal tissues of UC mice

**Figure 7:**
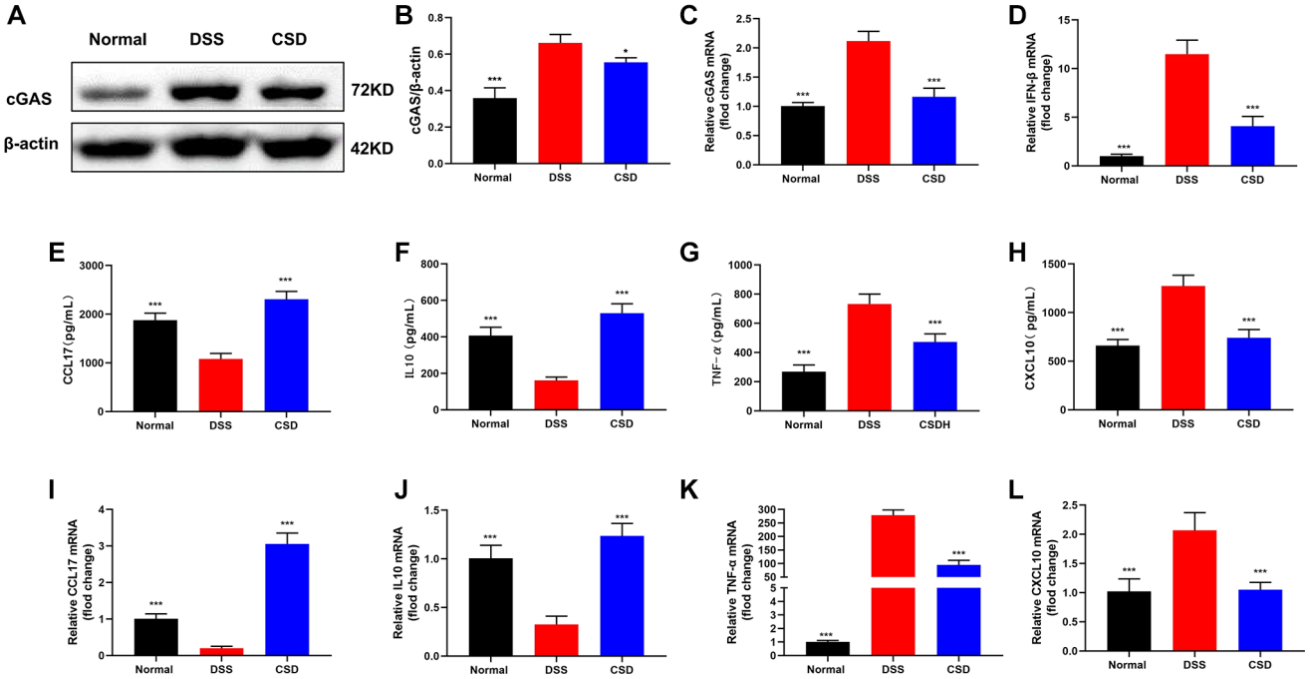
**CSD inhibited the expression of cGAS and the content of inflammatory factors in UC mice intestinal tissues.** (**A**) Representative WB of cGAS in the intestine; (**B**) Statistical map of cGAS protein content in the intestine; (**C**) cGAS mRNA levels in the intestine; (**D**) IFN-β mRNA levels in the intestine; (**E**) ELISA measured the content of CCL17 in the intestine; (**F**) ELISA measured the content of IL10 in the intestine; (**G**) ELISA measured the content of TNF-α in the intestine; (**H**) ELISA measured the content of CXCL10 in the intestine; (**I**) CCL17 mRNA levels in intestine; (**J**) IL10 mRNA levels in intestine; (**K**) TNF- α mRNA levels in intestine; (**L**) CXCL10 mRNA levels in intestine. The results are mean ± SD, *n* = 3–6, ^*^p < 0.05, ^***^p < 0.001 vs. DSS group.

As previously stated, ISD could activate cGAS to induce inflammatory tendencies in macrophages. Therefore, we were interested in whether DSS could influence changes in intestinal inflammation levels *in vivo* by inducing cGAS expression. WB and PCR results revealed that cGAS expression was increased in the DSS group compared to the control group, and the relative content of IFN-β mRNA was increased, which could significantly inhibit their expression after CSD administration (Figure 7A–7D). The levels of inflammation-related factors in the intestine were measured using ELISA and PCR. Compared with the normal group, CCL17, and IL10 expression decreased in the DSS group, and CSD treatment significantly increased their levels (Figure 7E, 7F, 7I, 7J). However, TNF-α and CXCL10 increased significantly after DSS induction, and CSD could significantly reduce this result (Figure 7G, 7H, 7K, 7L). These findings suggest that CSD could regulate DSS-induced macrophage polarization in UC mice by inhibiting cGAS and alleviating intestinal inflammation.

### CSD protects the intestinal barrier

**Figure 8:**
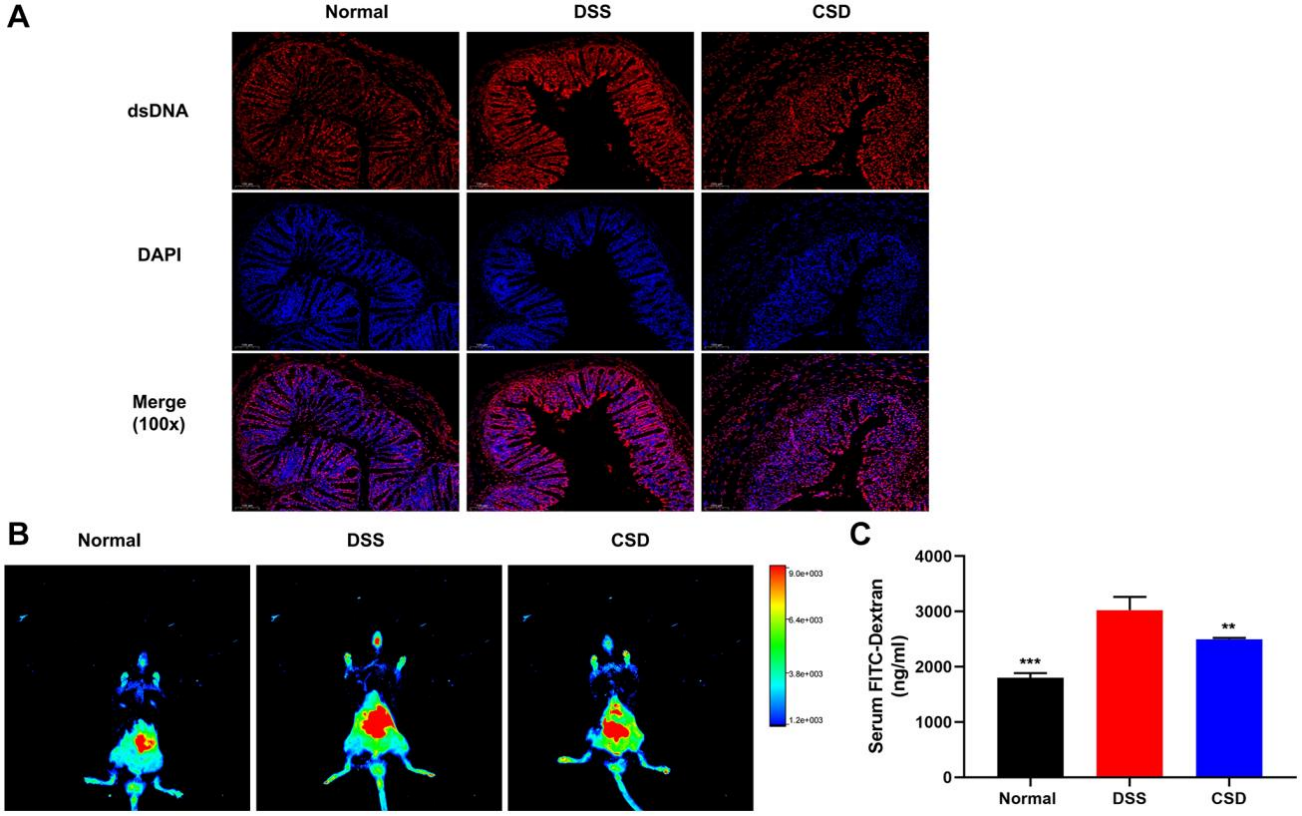
**CSD protected the intestinal barrier.** (**A**) Fluorescence expression of dsDNA in intestinal tissue, 100X magnification; (**B**) FITC-dextran distribution in the intestinal tract of mice; (**C**) Serum FITC-Dex levels. The results are mean ± SD, *n* = 3, ^**^p < 0.01, ^***^p < 0.001 vs. DSS group.

The dsDNA could activate cGAS, initiating IFN-β transcription [30]. Studies in DSS-induced UC mice revealed that the content of dsDNA in intestinal tissues increased, possibly due to DNA damage of intestinal epithelial cells (IEC) [32, 33]. We assumed that DSS-induced cGAS activation in UC mice was related to increased dsDNA after intestinal injury. Therefore, immunofluorescence was used to examine dsDNA expression in intestinal tissues. As expected, dsDNA expression was significantly higher in the DSS group than in the normal group and significantly down-regulated after CSD treatment (Figure 8A). This suggests that CSD may reduce DNA damage in intestinal cells, such as intestinal epithelial cells, which are important for protecting the intestinal barrier. Therefore, live imaging of small animals and the text of Fluorescein isothiocyanate-dextran (FITC-dex) in serum were used to detect intestinal barrier function in mice. In the DSS group, intestinal fluorescence intensity was higher than in the normal group, and serum FITC-dex content significantly increased. In contrast, it was down-regulated after CSD treatment (Figure 8B, 8C). These findings imply that CSD protects the intestinal barrier and may play a role in DNA repair in intestinal cells.

## DISCUSSION

The World Health Organization classifies UC as a modern refractory disease due to its complex etiology, difficulty of cure, easiness of relapse, and high risk of colon cancer [34]. The intervention targets of various ingredients are also diverse from the perspective of modern pharmacology. Therefore, the relevant targets and mechanisms of CSD’s therapeutic effect on UC must be investigated further for UC with a complex etiology.

Network pharmacology is widely used to identify potential drug targets and effect mechanisms. Network pharmacology was used in the study to predict potential targets and pathways of CSD in treating UC and the active components of CSD. The findings revealed that quercetin was one of the most effective components of CSD. Quercetin, a natural flavonoid, has an apparent anti-inflammatory effect by regulating macrophage polarization [8]. Many genes related to macrophage polarization were identified in the screened CSD targets for UC treatment, such as NOS2 (iNOS), CXCL10, TNF-α, IL-6, IL10, and IL-1β [8, 35]. Moreover, the biological functions of oxidative stress, lipopolysaccharide reaction, IL-17 signaling pathway, TNF-α, and other inflammation-related signaling pathways were enriched. These biological functions and inflammatory signaling pathways are associated with macrophage polarization.

Immune response disorder manifests as an imbalance in macrophage polarization, and it is one of the pathogenesis factors of UC, which is important in UC research. Numerous studies of inflammatory diseases indicate that early M1 cells accumulate at the site of inflammation to phagocyte bacteria and apoptotic cell debris, protecting tissues from invasion by foreign substances. However, excessive polarization of M1 cells causes serious tissue damage and an imbalance in the M1/M2 cell ratio, which results in more serious inflammatory factor storms [36]. The intestinal mucosa, the largest immune organ of the human body, contains many macrophages. Therefore, we studied the regulation of macrophage polarization by CSD using network pharmacology. We used RAW264.7 and BMDMs, two commonly used macrophages, *in vitro* studies. F4/80 and CD11b are surface markers of BMDMs, and the induced mature macrophages (CD11b^+^F4/80^+^) account for more than 95% of the total cells on the seventh day [28]. Our results indicate that this ratio reaches 99.9%, indicating successful isolation and induction of BMDMs.

Previous research has primarily used LPS and IFN-γ to stimulate M1 polarization and IL4/IL13 to stimulate M2 polarization [8, 37]. However, inflammation induced by LPS and IFN-γ is independent of cGAS [14], and ISD can activate cGAS and induce the production of IFN-β [38, 39]. Therefore, ISD, an exogenous dsDNA, was introduced here as a cellular inflammation model stimulator. iNOS is not expressed in macrophages in a physiological state but is often expressed after inflammatory stimulation. As a M1 macrophage marker, iNOS can produce NO, which is associated with DNA damage and cancer [40]. Arg1 is a M2 macrophage marker with tissue repair function that can catalyze the hydrolysis of L-arginine to ornithine and urea while reducing NO production [41]. CSD could effectively reverse the increase of iNOS and decrease in Arg1 caused by inflammation in RAW264.7 and intestinal tissues. M1 macrophages are usually labeled with CD11c, while M2 macrophages are labeled with CD206 [42]. CD11c^+^ macrophages were found close to the luminal surface, while CD206^+^ macrophages dominated the colonic macrophage pool and were located within the colonic lamina propria (CLP) and the base of intestinal crypts. Under inflammatory conditions, CD206^+^ and CD11c^+^ macrophages began to coexist throughout the CLP and at the muscularis mucosae membrane. M2/M1 is commonly used to indicate changes in macrophage polarization. M2/M1 decreased after ISD stimulation of successfully induced BMDMs and increased after CSD intervention. The results revealed that ISD could cause macrophage polarization imbalance, and CSD could regulate macrophage polarization imbalance *in vitro* and in the spleen, mesenteric lymph nodes, and intestine of UC mice.

The cGAS, a nucleic acid transferase family member, recognizes dsDNA using its DNA binding sites and zinc sac structure and can identify almost all types of dsDNA. The identification of free DNA in cells triggers a variety of signaling pathways, the most common of which is IFN-β activation by cGAS-stimulator of interferon genes (STING), and IFN-β cannot be activated when STING is absent or not expressed [43, 44]. cGAS activation was identified in the intestinal tissues of UC patients and mice [45], while another study demonstrated that cGAS mediated inflammation by acting as a macrophage polarization switch, promoting peritoneal macrophages and BMDMs to inflammatory phenotypes (M1). In previous proteomic studies, it was found that compared with the DSS model group, both CSD and its active ingredient quercetin down-regulated the expression of cGAS after treatment (Supplementary Figure 1). *In vivo*, the absence of cGAS reduces sepsis-induced acute lung injury by promoting the macrophage transformation from M1 to M2 phenotype [11]. Inhibiting the cGAS-STING signaling pathway can regulate the inflammatory response and polarization of pancreatic cancer tumor-associated macrophages, confirming that cGAS plays a role in macrophage polarization and inflammatory response [46]. Therefore, we speculated whether the CSD regulation of macrophage polarization is due to its involvement in cGAS-induced inflammatory response. CSD could reduce the expression of cGAS and inflammatory factors (IFN-β, CXCL10, and TNF-α) in macrophages while increasing the expression of anti-inflammatory factors (IL10 and CCL17) both *in vivo* and *in vitro*. The results revealed that ISD activated macrophage cGAS, while CSD reduced UC inflammation by inhibiting macrophage cGAS.

The increase in intestinal permeability is mainly due to the breakdown of the intestinal epithelial barrier, which is a key link in the pathogenesis of UC. Normal epithelium can keep harmful substances out of the intestine while effectively absorbing nutrients. However, permeability increases, and the opportunity for pathogens to invade the intestinal cavity increases, aggravating intestinal damage and inflammation [47]. Therefore, we determined the intestinal distribution of FITC-Dex and its content in serum after intragastric administration. We identified that FITC-Dex was widely distributed in the intestine after mold creation, with greater absorption into the blood and strong intestinal permeability, and CSD could alter the outcome. M2 is a macrophage with a tissue repair function, and the influence of its increased proportion on intestinal tissue repair is being investigated further. When intestinal inflammation occurs, pathogenic bacteria break the DNA double-strand of the host, causing host cell DNA damage and dsDNA accumulation [48]. Extracellular vesicles (EVs) from damaged colonic epithelium carry dsDNA to activate the STING pathway of macrophages and cause inflammation [36]. We identified that dsDNA expression increased significantly after CSD intervention while dsDNA content decreased in UC colon tissue. The results revealed that DSS induced intestinal DNA damage in UC mice, but CSD can reduce DNA damage and repair the intestinal barrier.

## CONCLUSION

In summary, the present study demonstrated that CSD could inhibit cGAS to restore M2/M1 ratio, repair UC intestinal damage, and reduce intestinal dsDNA content, thereby slowing UC progression. The study has enriched the relevant mechanism and provided experimental evidence to support CSD’s clinical treatment of UC.

## Supplementary Materials

Supplementary Figure 1

Supplementary Tables
