# Supplementary material for: Compound sophora decoction alleviates ulcerative colitis by regulating macrophage polarization through cGAS inhibition: network pharmacology and experimental validation
Source: Aging (Albany NY). 2024 Apr 10;16(8):6921–36. doi: 10.18632/aging.205734 (PMC11087132; doi:10.18632/aging.205734)
Supplement: Supplementary Figure 1 [file aging-16-205734-s001.pdf]

SUPPLEMENTARY FIGURE

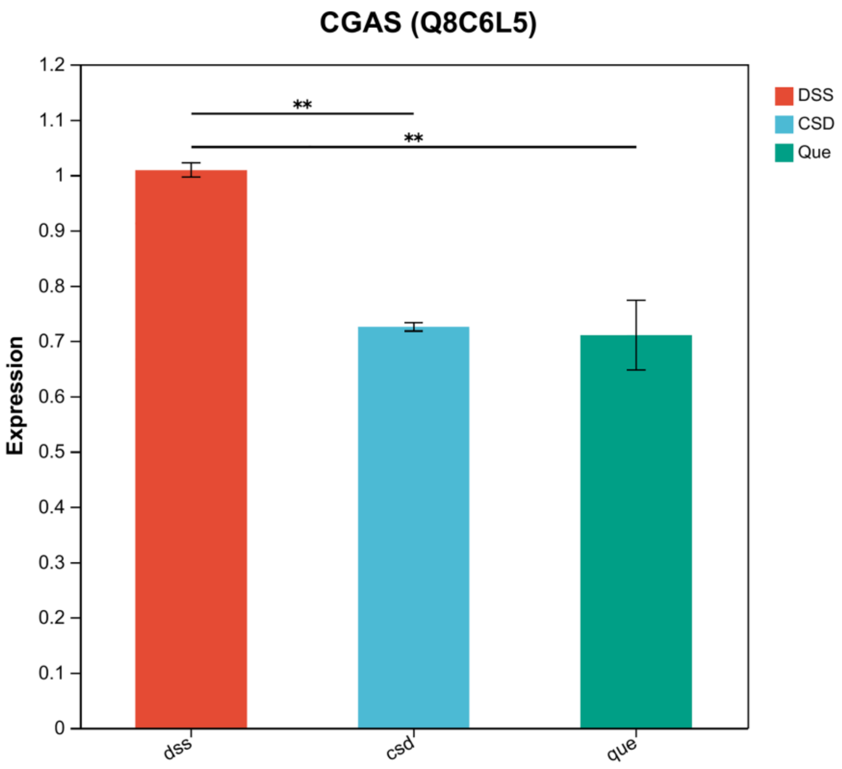

Supplementary Figure 1. Protein transcriptomics showed that both CSD and quercetin could significantly down-regulate cGAS protein in intestinal tissue of DSS induced UC mice. The results are mean ± SD, $^{**}p < 0.01$ vs. DSS group.
