# Supplementary material for: Compound sophora decoction alleviates ulcerative colitis by regulating macrophage polarization through cGAS inhibition: network pharmacology and experimental validation
Source: Aging (Albany NY). 2024 Apr 10;16(8):6921–36. doi: 10.18632/aging.205734 (PMC11087132; doi:10.18632/aging.205734)
Supplement: Supplementary Tables [file aging-16-205734-s002.pdf]

SUPPLEMENTARY TABLES

Supplementary Table 1. mRNA primers.

Gene		Primer Sequences (5′–3′)
ISD	Forward	TACAGATCTACTAGTGATCTATG
	Reverse	ACTGATCTGTACATGATCTACA
cGAS	Forward	TTCCACGAGGAAATCCGCTGAG
	Reverse	CAGCAGGGCTTCCTGGTTTTTC
iNOS	Forward	GAAGAAAACCCCTTGTGCTG
	Reverse	TCCAGGGATTCTGGAACATT
Arg1	Forward	CTTGGCTTGCTTCGGAACTC
	Reverse	GGAGAAGGCGTTTGCTTAGTTC
IFN-β	Forward	CTAACTGCAACCTTTCGAAGC
	Reverse	CTAGTGTCCTTTCATATGCAG
CCL17	Forward	CGAGAGTGCTGCCTGGATTACT
	Reverse	GGTCTGCACAGATGAGCTTGCC
IL10	Forward	GCTCTTACTGACTGGCATGAG
	Reverse	CGCAGCTCTAGGAGCATGTG
TNF-α	Forward	TACTGAACTTCGGGGTGATCG
	Reverse	TCCTCCACTTGGTGGTTTGC
CXCL10	Forward	CCAAGTGCTGCCGTCATTTTC
	Reverse	TCCCTATGGCCCTCATTCTCA
β-actin	Forward	AGACTTCGAGCAGGAGATGG
	Reverse	GCACTGTGTTGGCATAGAGG

Supplementary Table 2. Active components of CSD.

Mol ID	Molecule name	The drugs in CSD	OB (%)	DL
MOL000098	quercetin	*Sophora flavescens Aiton*, *Sanguisorba officinalis* L., *Panax notoginseng* (Burkill) F.H. Chen, *Glycyrrhiza glabra* L.	46.43	0.28
MOL000358	beta-sitosterol	*Sanguisorba officinalis* L., *Baphicacanthus cusia* (Nees) Bremek., *Panax notoginseng* (Burkill) F.H. Chen	36.91	0.75
MOL000422	kaempferol	*Sanguisorba officinalis* L., *Glycyrrhiza glabra* L.	41.88	0.24
MOL001484	Inermine	*Sophora flavescens Aiton*, *Glycyrrhiza glabra* L.	75.18	0.54
MOL004941	(2R)-7-hydroxy-2-(4-hydroxyphenyl) chroman-4-one	*Sophora flavescens Aiton*, *Glycyrrhiza glabra* L.	71.12	0.18
MOL000392	formononetin	*Sophora flavescens Aiton*, *Glycyrrhiza glabra* L.	69.67	0.21
MOL000211	Mairin	Sanguisorba officinalis L., Glycyrrhiza glabra L.	55.38	0.78
MOL001792	DFV	*Panax notoginseng* (Burkill) F.H. Chen, *Glycyrrhiza glabra* L.	32.76	0.18
